# Locally Acquired *mcr-1* in *Escherichia coli*, Australia, 2011 and 2013

**DOI:** 10.3201/eid2307.161638

**Published:** 2017-07

**Authors:** Justin A. Ellem, Andrew N. Ginn, Sharon C.-A. Chen, John Ferguson, Sally R. Partridge, Jonathan R. Iredell

**Affiliations:** Westmead Hospital, Westmead, New South Wales, Australia (J.A. Ellem, A.N. Ginn, S.C.-A. Chen, S.R. Partridge, J.R Iredell);; New South Wales Health Pathology, Westmead (J.A. Ellem, A.N. Ginn, S.C.-A. Chen, J.R. Iredell);; The University of Sydney, Sydney, New South Wales, Australia (A.N. Ginn, S.C.-A. Chen, S.R. Partridge, J.R. Iredell);; The Westmead Institute for Medical Research, Westmead (A.N. Ginn, S.R. Partridge, J.R. Iredell);; John Hunter Hospital, Newcastle, New South Wales, Australia (J. Ferguson);; Pathology North, Newcastle (J. Ferguson); University of Newcastle, Newcastle (J. Ferguson)

**Keywords:** antimicrobial resistance, colistin, Escherichia coli, plasmid, mcr-1, IncI2 plasmid, bacteria, colistin-resistant Enterobacteriaceae, importation events, Australia

## Abstract

We identified discrete importation events of the *mcr-1* gene on incompatibility group IncI2 plasmids in *Escherichia coli* isolated from patients in New South Wales, Australia, in 2011 and 2013. *mcr-1* is present in a small minority of colistin-resistant Enterobacteriaceae and appears not to be established locally.

The *mcr-1* (mobile colistin resistance) gene was discovered in *Escherichia coli* isolates collected during 2011–2014 from animals and meat products and from *Klebsiella pneumoniae* from human patients in China (*1*). Since then, *mcr-1* has been identified on plasmids of various incompatibility (Inc) types associated with 0, 1, or 2 copies of the insertion sequence (IS) IS*Apl1* (*2*), and it has been identified in other species (most notably *Salmonella* spp.) and in several other countries (*3*), not including Australia. We analyzed colistin resistance in *Enterobacteriaceae* isolates collected in Sydney, New South Wales, Australia, during 2007–2016.

## The Study

We reviewed 4,555 isolates of the family *Enterobacteriaceae* from 2007–2016 that were available for further testing for colistin (polymyxin E) resistance, excluding species that are intrinsically resistant. These isolates were from specimens tested at or referred to the Centre for Infectious Diseases Microbiology Laboratory Services at Westmead Hospital, Sydney, New South Wales, Australia, and were all resistant to third-generation cephalosporins, carbapenems, or both. For antimicrobial drug susceptibility testing, we used the Phoenix Automated Microbiology System (panels NMIC/ID-80, NMIC/ID-101, and NMIC-203; Becton Dickinson, Franklin Lakes, NJ, USA). Of the 4,555 isolates, 96 (2.1%) had a colistin (polymyxin E) minimum inhibitory concentration (MIC) of >2 μg/mL, which corresponds to EUCAST (European Committee on Antimicrobial Susceptibility Testing) breakpoints (http://www.eucast.org/clinical_breakpoints/). The 96 colistin-resistant isolates consisted of 44 *K. pneumoniae*, 8 *K. oxytoca*, 18 *E. coli*, 19 *Enterobacter* spp., 5 *Hafnia alvei*, and 2 *Citrobacter freundii* isolates. By using published primers (*1*), we identified *mcr-1* in 2 of the colistin-resistant *E. coli* isolates; these isolates were from patients in different cities in New South Wales.

In September 2011, *E. coli* JIE2288 (colistin MIC of 4 μg/mL) was isolated from the urine of a 70-year-old woman after she had been in the intensive care unit (ICU) at Westmead Hospital, a large metropolitan hospital, for 2 months for management of a subarachnoid hemorrhage. The woman was not administered colistin/polymyxin antimicrobial drugs during hospitalization. The *E. coli* JIE2288 isolate was also resistant to amikacin, gentamicin, tobramycin, amoxicillin/clavulanic acid, cefotaxime, ceftazidime, cefepime, cefoxitin, trimethoprim/sulfamethoxazole, and ciprofloxacin, according to EUCAST breakpoints. The isolate was susceptible to piperacillin/tazobactam (MIC of <4/4 μg/mL) and meropenem (MIC of <1 μg/mL). The patient had no history of overseas travel in the previous 5 years, and while she was in the ICU, no other ICU patients had a recognized colistin-resistant infection and none were receiving colistin treatment. The patient was housed in a single room, and standard infection-control precautions (disposable gown and gloves) were used. The patient was treated with a course of meropenem, and subsequent cultures of her urine did not contain colistin-resistant *E. coli*.

In July 2013, a second colistin-resistant *E. coli* strain (herein designated JIE3685; colistin MIC of >4 μg/mL) was referred to our laboratory after being isolated from the urine of a 71-year-old woman with diabetes mellitus. The woman had sought care from a community physician in Newcastle, New South Wales, for a urinary tract infection. She had had no healthcare contact in the previous 2 years and no history of travel outside Australia in the previous 5 years. JIE3685 was also resistant to cefotaxime, cefepime, ciprofloxacin, gentamicin, tobramycin, and trimethoprim/sulfamethoxazole, but it was susceptible to amoxicillin/clavulanic acid, ceftazidime, piperacillin/tazobactam, meropenem, and amikacin.

We obtained colistin-resistant transconjugants from *E. coli* JIE2288 and JIE3685 essentially as previously described (*4*) and, using PCR, confirmed that they carried *mcr-1*. We subjected DNA prepared from the original JIE2288 and JIE3685 isolates and transconjugants to paired-end sequencing (NextSeq 500 platform; Illumina, San Diego, CA, USA) and assembled the sequences by using SPAdes 3.7.1 (http://bioinf.spbau.ru/spades). Using PlasmidFinder (https://cge.cbs.dtu.dk/services/PlasmidFinder/), we identified IncI2 replicons in both isolates, and using the MLST tool on the Centre for Genomic Epidemiology website (https://cge.cbs.dtu.dk/services/MLST/), we identified JIE2288 as sequence type (ST) 167 (clonal complex [CC] 10) and JIE3865 as ST93 (CC168). Other ST167 isolates carrying *mcr-1* have been reported; for example, *mcr-1* was carried on a ≈65-kb IncI2 plasmid and the *bla*_NDM-9_ gene on a different plasmid in an isolate from chicken meat in China in 2014 (*5*). Furthermore, ST93 (CC168) is a known zoonotic pathogenic strain previously associated with transmission of *mcr-1* from animals to humans in Laos (*6*) and China (*7*).

Most of the IncI2 plasmid from *E. coli* JIE2288 that carries *mcr-1* (designated pJIE2288-1) was assembled as a single contig, missing only the shufflon region that rearranges to change the end of the *pilV* gene (*8*), causing assembly issues that may result in apparent differences between plasmids carrying *mcr-1* (*9*). We obtained the shufflon segments of pJIE2288-1 by mapping raw reads to individual shufflon segments from the archetypal IncI2 plasmid R721 (GenBank accession no. AP002527) and then assembled them in the same order as in R721 to generate a complete plasmid sequence (*8*). An additional plasmid, carrying the *bla*_CTX-M-14a_ gene (IncI1, designated pJIE2288-2), was identified in JIE2288. BLAST (https://blast.ncbi.nlm.nih.gov/Blast.cgi) searches with 2 contigs from JIE3685 identified several closely matching plasmids (Table). We used pEC5-1, which had been sequenced by long-read methods, as a reference to resolve a repeated region and as a template for the shufflon, thus enabling complete assembly of a plasmid designated pJIE3685-1. We submitted the sequences of pJIE2288-1 and pJIE3685-1 to GenBank under accession numbers KY795977 and KY795978, respectively.

**Table:** IncI2 plasmids that carry *mcr-1* and are closely related to pJIE2288-1 or pJIE3685-1 from *Escherichia coli* isolates from separate patients in New South Wales, Australia, 2011 and 2013*

Study plasmid, related plasmids	GenBank accession no.	Genus and species	Source	Country	Year	Insertion sequence
pJIE2288-1	KY795977	*E. coli*	Human urine	Australia	2011	–
pEG430-1	LT174530	*Shigella sonnei*	Human feces	Vietnam	2008	*Apl1*
pHSSH22-MCR1	KX856067	*Salmonella* Typhimurium	Human feces	China	2012	
pHSSH23-MCR1	KX856068	*Salmonella* Enteritidis	Human feces	China	2012	*Apl1*
pHNSHP45	KP347127	*E. coli*	Pig farm	China	2013	*Apl1*, *683*
pHeNE867†	KU934208	*E. coli*	Chicken	China	2013	–
pABC149-MCR-1	KX013538	*E. coli*	Human blood	UAE	2013	*Apl1*
pEc_04HAE12	KX592672	*E. coli*	Human blood	China	2014	*1*
pR150626	KY120366	*Salmonella* Typhimurium	Human	Taiwan		–
pEC006	KY471144	*E. coli*		South Korea‡		*150*
pJIE3685-1	KY795978	*E. coli*	Human urine	Australia	2011	–
pEC5-1	CP016185	*E. coli*	Chicken liver	Malaysia	2013	*1*
pEC13-1	CP016186	*E. coli*	Pond water	Malaysia	2013	–
pS2.14-2	CP016187	*E. coli*	Chicken feed	Malaysia	2013	–
pEc_20COE13§	KY012274	*E. coli*	Human blood	China	2014	–
pBA77-MCR-1	KX013539	*E. coli*	Human urine	Bahrain	2015	*1294*
pP111	KY120365	*Salmonella* Typhimurium	Pig	Taiwan		–

*UAE, United Arab Emirates; –,no known insertion sequences were identified. Blank cells indicate that the information is not available. †Carries *mcr-1.3* variant (AA111–2GG) encoding MCR-1.3 with a single (Ile38Val) change from MCR-1 (*10*). ‡This information is assumed from a GenBank entry rather than from a published report. §This plasmid has many missing or extra nucleotides in homopolymeric regions compared with all other related plasmids.

pJIE2288-1 and pJIE3685-1 each carry *mcr-1* as the only resistance gene (located in the same position), and both lack IS*Apl1*, but they do have differences in the remainder of their IncI2 backbones. pJIE2288-1 (60.733 kb) is closely related to 9 other sequenced *mcr-1* plasmids (differing by only a few single nucleotide changes in backbone regions), some of which carry IS*Apl1* upstream of *mcr-1*, another IS, or both (Table). pJIE3685-1 (60.960 kb) is closely related to 6 other IncI2 *mcr-1* plasmids (again, differing by only a few single nucleotide changes), all of which also lack IS*Apl1* (Table).

We noted differences between pJIE2288-1 and pJIE3685-1 in the *mcr-1* promoter (*11*) and putative ribosome binding site and these or other differences in this region in available *mcr-1* sequences (Figure). We examined the chromosomal *arnBCADTEF*, *pmrAB*, and *phoPQ* genes of each isolate but did not identify any of the changes previously reported in association with colistin resistance in *E. coli* (*12*). This finding suggests that *mcr-1* may be the sole phenotype determinant in these 2 strains.

**Figure:**
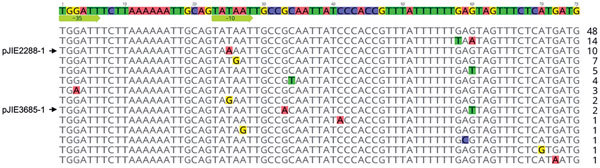
Differences in promoter and ribosome binding site regions of *mcr-1* in plasmids from *Escherichia coli* in Australia (indicated by arrows) and in other sequences available from GenBank. The sequences end with the ATG start codon of *mcr-1* and a second ATG codon that follows it. The −35 and −10 regions of the proposed promoter (*11*) are indicated by arrows. The numbers to the right indicate how many times each variant has been seen among available sequences.

## Conclusions

We describe 2 *E. coli* isolates collected from patients in different cities in Australia ≈2 years apart that belong to STs previously associated with *mcr-1* and which carry this gene on plasmids that are virtually identical to previously described *mcr-1*–bearing plasmids from Asia and the Middle East. Multiple importations of resistant isolates into the community microflora are to be expected, and these 2 case-patients in Australia had no history of travel outside Australia or direct links to each other. Australia’s regional neighbors use colistin extensively in agriculture (*13*), but in Australia, its usage in agriculture and healthcare is minimal, and neither colistin nor polymyxin drugs appear in the top 20 antimicrobial drugs prescribed in Australia (*14*). Notwithstanding the limitations of this opportunistic study, our findings indicate that colistin resistance is unusual among Enterobacteriaceae in Australia and that *mcr-1* is neither a key mechanism nor yet widely disseminated in this country despite multiple importation events.
